# Early recognition of *CLN3* disease facilitated by visual electrophysiology and multimodal imaging

**DOI:** 10.1007/s10633-023-09930-1

**Published:** 2023-03-25

**Authors:** Dhimas H. Sakti, Elisa E. Cornish, Clare L. Fraser, Benjamin M. Nash, Trent M. Sandercoe, Michael M. Jones, Neil A. Rowe, Robyn V. Jamieson, Alexandra M. Johnson, John R. Grigg

**Affiliations:** 1 Save Sight Institute, Speciality of Clinical Ophthalmology and Eye Health, Faculty of Medicine and Health, The University of Sydney, Sydney Eye Hospital Campus, 8 Macquarie St, Sydney, NSW 2001 Australia; 2 Eye Genetics Research Unit, The Children’s Hospital at Westmead, Save Sight Institute, Children’s Medical Research Institute, The University of Sydney, Sydney, NSW Australia; 3 Sydney Genome Diagnostics, Sydney Children’s Hospital Network (Westmead), Sydney, Australia; 4 Department of Ophthalmology, Faculty of Medicine, Public Health, and Nursing, Universitas Gadjah Mada, Yogyakarta, Indonesia; 5 Department of Ophthalmology, Sydney Children’s Hospital Network (Westmead), Sydney, Australia; 6 Department of Neurology, Sydney Children’s Hospital, University of New South Wales, Sydney, Australia

**Keywords:** Batten, CLN3, Neuronal ceroid lipofuscinoses, Lysosomal storage disorders

## Abstract

**Background:**

Neuronal ceroid lipofuscinosis is a group of neurodegenerative disorders with varying visual dysfunction. *CLN3* is a subtype which commonly presents with visual decline. Visual symptomatology can be indistinct making early diagnosis difficult. This study reports ocular biomarkers of *CLN3* patients to assist clinicians in early diagnosis, disease monitoring, and future therapy.

**Methods:**

Retrospective review of 5 confirmed *CLN3* patients in our eye clinic. Best corrected visual acuity (BCVA), electroretinogram (ERG), ultra-widefield (UWF) fundus photography and fundus autofluorescence (FAF), and optical coherence tomography (OCT) studies were undertaken.

**Results:**

Five unrelated children, 4 females and 1 male, with median age of 6.2 years (4.6–11.7) at first assessment were investigated at the clinic from 2016 to 2021. Four homozygous and one heterozygous pathogenic *CLN3* variants were found. Best corrected visual acuities (BCVAs) ranged from 0.18 to 0.88 logMAR at first presentation. Electronegative ERGs were identified in all patients. Bull’s eye maculopathies found in all patients. Hyper-autofluorescence ring surrounding hypo-autofluorescence fovea on FAF was found. Foveal ellipsoid zone (EZ) disruptions were found in all patients with additional inner and outer retinal microcystic changes in one patient. Neurological problems noted included autism, anxiety, motor dyspraxia, behavioural issue, and psychomotor regression.

**Conclusions:**

*CLN3* patients presented at median age 6.2 years with visual decline. Early onset maculopathy with an electronegative ERG and variable cognitive and motor decline should prompt further investigations including neuropaediatric evaluation and genetic assessment for *CLN3* disease. The structural parameters such as EZ and FAF will facilitate ocular monitoring.

## Introduction

The neuronal ceroid lipofuscinoses (NCLs) are a group of autosomal recessive lysosomal storage disorders (LSD) and together are one of the most frequent causes of neurodegenerative disease in children. The incidence of NCL ranges from 0.1 to 8 per 100,000 live births [1–7]. Isolated retinal *CLN3* disease accounted for 1% of all inherited retinal disease (IRD) in a French cohort [8]. There are a number of recent publications reporting isolated retinal findings in patients with CLN3 mutations [9–11]. Analysis of these reports suggests that it is more likely that the 1 kb homozygous deletion is associated with the syndromic *CLN3* phenotype, while compound heterozygous mutations are more likely to be found in the isolated retinal degeneration phenotype. NCL patients experience myoclonic seizures, progressive visual deterioration, cognitive dysfunction, motor decline, and premature death [11–13]. These clinical features often present asynchronously, making diagnosis difficult and often delayed. Classically, NCL was classified based on age at onset (congenital, infantile, late infantile, juvenile, and adult). To date, 13 causative genes have been identified (*CLN1* to *8* and *CLN10* to *14*) with *CLN3* being the most prevalent cause [11, 12, 14].

*CLN3* disease was formerly known as ‘juvenile neuronal ceroid lipofuscinosis’ (JNCL) and can initially present as with isolated visual symptoms or with progressive neurological dysfunction. Wang et al. reported that the *CLN3* associated visual symptoms can exhibit rod-cone or cone-rod dystrophy (RCD or CRD) phenotype [15]. In that study, 5 patients from a total of 123 retinal degeneration patients had a *CLN3* mutation with 4 RCD and 1 CRD phenotype [15]. Data from our previous study showed that all *CLN3* patients in our study centre had an electronegative ERG, suggesting its importance in this particular diagnosis [16].

CLN3 is a lysosomal membrane protein involved with glycosylation and phosphorylation at several sites, with localization to synaptic compartments in neuronal cells. This localization might suggest a distinctive role of the CLN3 protein within neurons that makes the central nervous system (CNS) susceptible in this disease [17].

Understanding of the ophthalmological findings is crucial to early diagnosis of *CLN3-*related disease, as these commonly precede the development of neurological signs, with retinal examination using multimodal imaging frequently identifying bull’s eye maculopathy, optic disc pallor, and/or bone spicule formation. These structural findings overlap with Stargardt disease or retinitis pigmentosa (RP) [18, 19]. Where *CLN3* disease is a differential diagnosis, it is critical that a full-field electroretinogram (ffERG) is performed, as this may demonstrate an electronegative ERG (b:a ratio ≤ 1 in dark adapted 3.0 or 12.0 ERG) [16, 18, 20–22]. Other classical ocular features of *CLN3* disease may then be elucidated on ophthalmic examination, alerting the clinician to the possibility of this disorder and the need for neurogenetic review.

Novel therapies for *CLN3*-related disease are currently emerging into clinical trials. Early diagnosis is therefore vital to increase the possibility of administering a novel *CLN3* disease therapy at a time when maximal benefit might be achieved. Ocular biomarkers become challenging to obtain as neurological deterioration progress. The purpose of this study is to report ocular findings of *CLN3* disease patients to aid early diagnosis, enable disease monitoring, and assist further trials of novel CLN3 therapies.

## Methods

Retrospective evaluation of 5 confirmed *CLN3* disease patients in our tertiary referral clinic were included in this study. They were referred for ophthalmic review and subsequently underwent single genetic testing for *CLN3* disease. The age when the patients were referred and the age of ocular and neurological onset were recorded. Age of ocular and neurological onset was determined by the earliest time point of reported ocular and neurological symptoms. Best corrected visual acuity (BCVA), retinal imaging, spectral domain-optical coherence tomography (SD-OCT), and full-field electroretinogram (ffERG) data were reviewed at baseline (BL) and follow-up (FU). BCVA was measured using a logarithm of minimum angle of resolution (logMAR). Patients with BCVA worse than 1.0 logMAR (6/60 on Snellen) were examined using Sheridan-Gardiner single letter and if failed this continued to finger counting, hand movement, and perception of light. BCVA values were then converted to logMAR equivalent values as described by Lange et al. [23, 24].

The study followed the tenets of the Declaration of Helsinki and was approved by the local ethical committee. Disease severity was calculated using the recently described Hamburg *CLN3* Ophthalmic Rating Scale [25]. This scale consists of visual acuity, fundus, and OCT score with maximum points of 14. The scale then is translated into *CLN3* grades of grade 0 (unaffected) = 14 points, grade 1 (affected) = 10–13 points, grade 2 (severely affected) = 5–9 points, and grade 3 (end stage) = 0–4 points.

### Retinal imaging

Ultra-widefield (UWF) fundus pseudocolour imaging and UWF-fundus autofluorescence (UWF-FAF) were performed using the Optos system (Optos plc, Dunfermline, UK).

### Spectral domain-optical coherence tomography (SD-OCT)

SD-OCT imaging was acquired using the Heidelberg Spectralis (Heidelberg Engineering, Germany) and Zeiss Cirrus (Carl Zeiss Meditec, Dublin, CA, USA). Retinal layers and central macular thickness were examined. Bruch membrane and internal limiting membrane markers were manually adjusted to ensure precision in measuring retinal thickness. Central subfield thickness (CST) and central macular thickness (CMT) are both commonly used terms in ophthalmology to describe the thickness of the central retina. Central subfield thickness (CST), also known as foveal thickness, was defined as the average thickness of the central 1 mm subfield centred at the fovea on ETDRS grid [26].

### Electrophysiology

Testing strategies included pattern ERG (pERG) and full-field ERG (ffERG) using Espion (Diagnosys, Lowell, Massachusetts USA). Visual electrophysiology was performed according to International Society for Clinical Electrophysiology of Vision (ISCEV) standards [27–29]. Gold foil; Dawson, Trick, and Litzkow (DTL); or skin electrodes were used depending on the level of patient’s cooperation. Paediatric non-standard abbreviated ERG protocol was done using a modified Great Ormond Street Hospital (GOSH) protocol utilizing handheld Grass (Gr) strobe for the most uncooperative patient [28, 30]. Pulse period 2 (2/s) and flicker Gr intensity 1 (Gr1) were used instead of 3/s and Gr4, respectively. The b:a wave ratio was calculated from dark adapted (DA) ffERG 3.0 or 12.0 with a value of ≤ 1.0 defined as an electronegative ERG [16, 31].

## Results

Five unrelated children with biallelic *CLN3* pathogenic variants were included in the study, 4 females and 1 male with median age at referral of 6.2 (4.6–11.7) years (yrs). Median age at ocular onset was 5.1 (2.6–11.6) yrs with P1 who had the earliest ocular onset while P5 the latest. Two patients (P1 and P2) had FU data. Hamburg *CLN3* ophthalmic rating scale at BL was ranging from 9 (affected) to 13 (severely affected) (Table 1). P1 progressed from affected to end stage while P2 from affected to severely affected.

**Table 1:** Characteristic of *CLN3* study patients

Characteristics				P1	P2	P3	P4	P5
Age at BL (years)/sex				4.6/F	5.9/F	6.2/M	7.1/F	11.7/F
Ancestry				Caucasian	Caucasian	Caucasian	Caucasian	South Asian
Ocular onset				2 years before BL	2 years before BL	4 months before BL	2 years before BL	1 month before BL
Ocular onset age (years)				2.6	3.9	5.9	5.1	11.6
Presenting symptoms				Hold objects closer	Blinks a lot, Colour vision concerns	Sits closer to television	Hold objects closer	Reduced distance VA
Neurological problems				Autism	Speech delay, significant anxiety	Behavioural abnormality suspect	Motor dyspraxia, behavioural issue (biting friends)	Not reported to date
Neurological problems onset age (years)				4.6	4.9	1.9	not clear	NA
Interval of neurological from ocular onset (year), before/after ocular onset				2, after ocular onset	1, after ocular onset	4, before ocular onset	not clear	NA
BL BCVA (LogMAR)	RE			0.18	0.6	0.88	0.54	0.5
	LE			0.4	0.38	0.88	0.56	0.56
Refractive correction (SE)	RE			3.75	1.5	0.625	0.5	-2.75
	LE			NA	1.875	1.375	-0.375	-2.5
FU duration (years)				2.2	5.6	NA	NA	NA
FU BCVA (LogMAR)	RE			2.7	2.7	NA	NA	NA
	LE			2.7	2.7	NA	NA	NA
Fundus picture (age in years)	BL			BEM, macular yellow-orange appearance (4.6)	BEM (5.9)	BEM, macular yellow-orange appearance, vessel rarefication, retinal atrophy outside vascular arcade (6.2)	BEM, vessel rarefication (7.1)	BEM, macular striae, macular yellow-orange appearance, vessel rarefication (11.7)
	FU			Macular yellow-orange appearance, vessel rarefication, retinal atrophy outside vascular arcade (6.8)	BEM, macular yellow-orange appearance, vessel rarefication, retinal atrophy on inferonasal area (9.4)	NA	NA	NA
FAF (age in years)	BL			HyperAF ring surrounding hypoAF fovea (4.6)	HyperAF ring surrounding hypoAF fovea (5.9)	HyperAF ring surrounding hypoAF fovea, hypoAF corresponding to retinal atrophy (6.2)	hyperAF ring surrounding hypoAF fovea (7.1)	hyperAF ring surrounding hypoAF fovea (11.7)
	1^st^ FU			More apparent perifoveal hyperAF ring (5.3)	More apparent perifoveal hyperAF ring (7)	NA	NA	NA
	2^nd^ FU			Perifoveal hyperAF ring disappearing, hypoAF outside vascular arcade corresponding to retinal atrophy (6.8)	Perifoveal hyperAF ring disappearing, replaced by hypoAF ring. HypoAF on inferonasal area corresponding to retinal atrophy (9.4)	NA	NA	NA
Macular OCT (age in years)	BL			EZ loss on fovea (4.6)	EZ loss on fovea (5.9)	EZ loss on fovea (6.2)	EZ loss on fovea (7.1)	Schitic changes on macula and EZ loss on fovea (11.7)
	FU			Complete EZ loss (6.3)	Complete EZ loss (9.4)	NA	NA	NA
CST (μM)	RE			104	134	112	101	145
	LE			107	125	118	99	111
IR				BEM	BEM	BEM	BEM	macular striae
Hamburg CLN3 ophthalmic rating scale*	BL scale (age in years)			11 (4.6)	13 (5.9)	10 (6.2)	9 (7.1)	9 (11.7)
	BL grade			1 (affected)	1 (affected)	1 (affected)	2 (severely affected)	2 (severely affected)
	FU scale (age in years)			3 (6.8)	5 (9.4)	NA	NA	NA
	FU grade			3 (end stage)	2 (severely affected)	NA	NA	NA
ffERG or flash ERG (age in years)	BL			Skin electrode. Electronegative waveform, significantly attenuated scotopic and photopic ERG with some residual signals. (4.6)	Skin electrode. Electronegative waveform, red stimulation under dark adapted conditions shows presence of rod but difficult to find cone component responses. (5.9)	DTL electrode. Significantly reduced scotopic signals and marked electronegative waveform, significantly reduced photopic signals. (6.3)	Paediatric non-standard abbreviated ERG protocol using skin electrode. Electronegative waveform with cone dysfunction. (7.1)	Gold foil electrode. Broadened and delayed DA ERG, electronegative waveform, peak time delay and bifid waveform in 30 Hz flicker, reduced and delayed LA 3.0, reduced on and off bipolar responses. (11.8)
	FU			NA	Scotopic electronegative, significantly reduced and delayed photopic response. (7)	NA	NA	NA
b:a ratio	BL	RE	DA 3.0	0.6	0.7	0.6	0.9	0.8
			DA 12.0	0.7	0.8	0	0.9	0.6
		LE	DA 3.0	0.7	0.6	0.4	1.7	0.8
			DA 12.0	0.7	0.7	0.5	1.3	0.8
	FU	RE	DA 3.0	NA	0.5	NA	NA	NA
			DA 12.0		0.7			
		LE	DA 3.0		0.5			
			DA 12.0		0.8			
pERG				Undetectable	Almost undetectable	15° are essentially undetectable, minimal rise in signals to the 30° field	Noisy	15° was normal but failure of doubling in 30°
Genetic variant				*CLN3*	*CLN3*	*CLN3*	*CLN3*	*CLN3*
Allele 1				c.461-280_677 + 382del	c.461-280_677 + 382del	c.461-280_677 + 382del	c.461-280_677 + 382del	c.461-280_677 + 382del
Allele 2				c.461-280_677 + 382del	c.461-280_677 + 382del	c.461-280_677 + 382del	c.461-280_677 + 382del	c.680A > G p.(Tyr227Cys)

^*^Hamburg  CLN3 ophthalmic rating scale 14 = grade 0 (unaffected), 10–13 = grade 1 (affected), 5–9 = grade 2 (severely affected), 0–4 = grade 3 (end stage) [21]

A novel missense variant (P5), c.680A > G *p*.(Tyr227Cys) was classified as likely pathogenic according to the ACMG classification (is present in low frequency (0.0004%) in the control population gnomAD, variant is in trans (separate alleles) with a confirmed pathogenic variant, all *in silico* tools predict a deleterious effect on the gene or gene product, and patient’s phenotype is highly specific for a gene with a single genetic aetiology)

*ACMG* American College of Medical Genetics, *AF* autofluorescence, *BL* baseline, *BCVA* best corrected visual acuity, *BEM* Bull’s eye maculopathy, *CST* central subfield thickness, *DA* dark adapted, *ERG* electroretinogram, *EZ* ellipsoid zone, *ffERG* full-field electroretinogram, *FU* follow-up, *IR* infra-red, *LA* light adapted, *LE* left eye, *pERG* pattern electroretinogram, *RE* right eye, *NA* not applicable. Variant is named according to the NM_001042432.1 transcript reference sequence

### Genetics and pathology investigations

The cohort in this study consisted of 4 patients with a previously reported homozygous pathogenic *CLN3* variant (P1-P4) and one patient with compound heterozygous *CLN3* variants (P5) (Table 1). The recurrent pathogenic variant *CLN3*: c.461-280_677 + 382del was identified in all 5 patients investigated. Only P3 underwent peripheral blood film microscopy and electron microscopy both with positive result of vacuolated lymphocytes and fingerprint inclusions, respectively.

#### BCVA

**Fig. 1:**
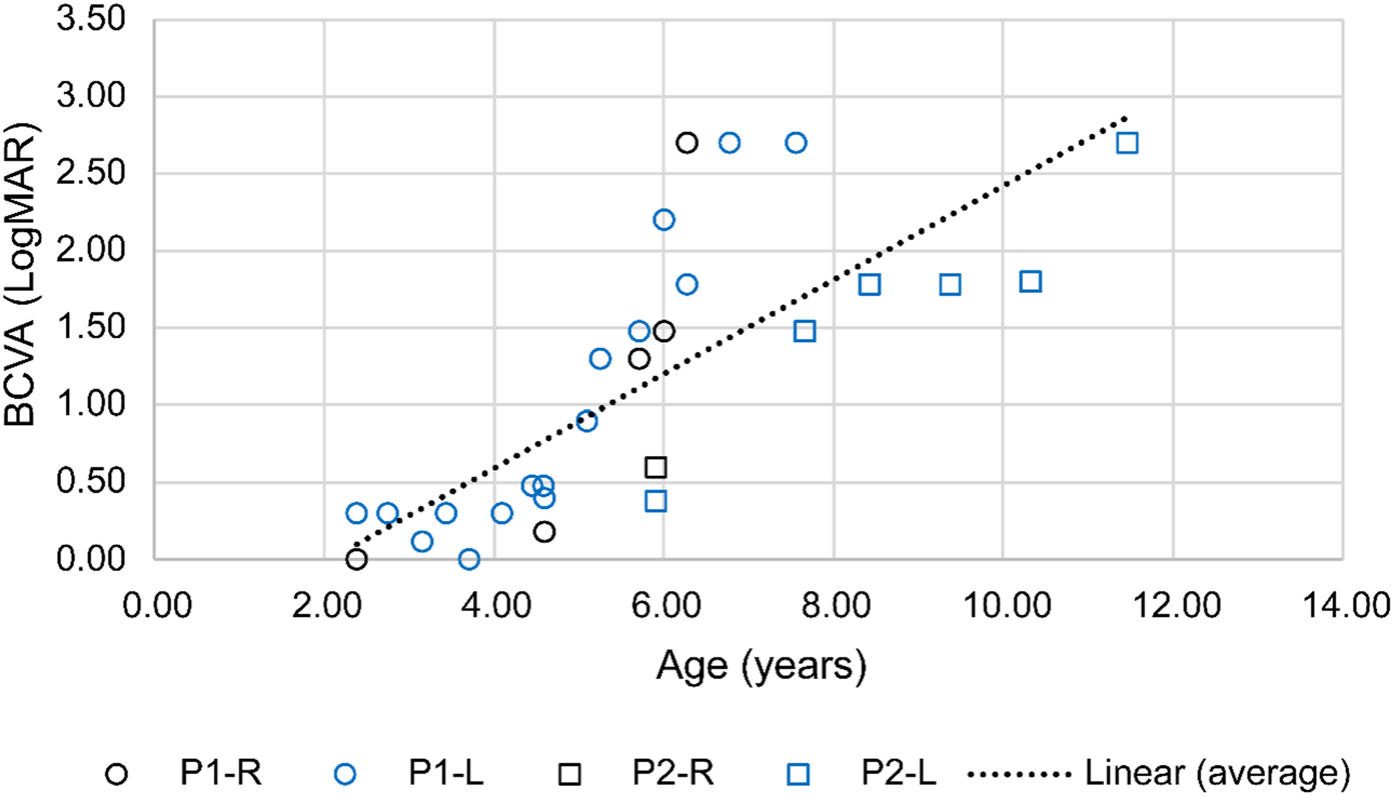
BCVA relationship with age. BCVA was plotted against age for patients P1 & P2. The BCVA trend deteriorated with increasing age. P1 eyes are shown in circle. P2 eyes are shown in square. Right eyes are presented in black colour, while left eyes in blue colour. *BCVA* Best corrected visual acuity

BCVAs ranged from 0.18 to 0.88 logMAR at BL with follow-up (FU) obtainable from 2 patients (P1 and P2) (Table 1). These 2 patients (4 eyes) had an average of 0.75 (0.41) logMAR loss per year during an average of 3.9 (2) years of FU and worst eventual FU BCVA (2.7 logMAR). P1 had earlier ocular onset than P2 and thus had the worst BCVA (2.7 logMAR) at an earlier age (6.8 vs 11.5 yrs) (Fig. 1). At the point of similar age (11–12 yrs), P2 had far worse BCVA than P3 (2.7 logMAR vs 0.5&0.56 logMAR) (Fig. 1).

### Retinal imaging

**Fig. 2:**
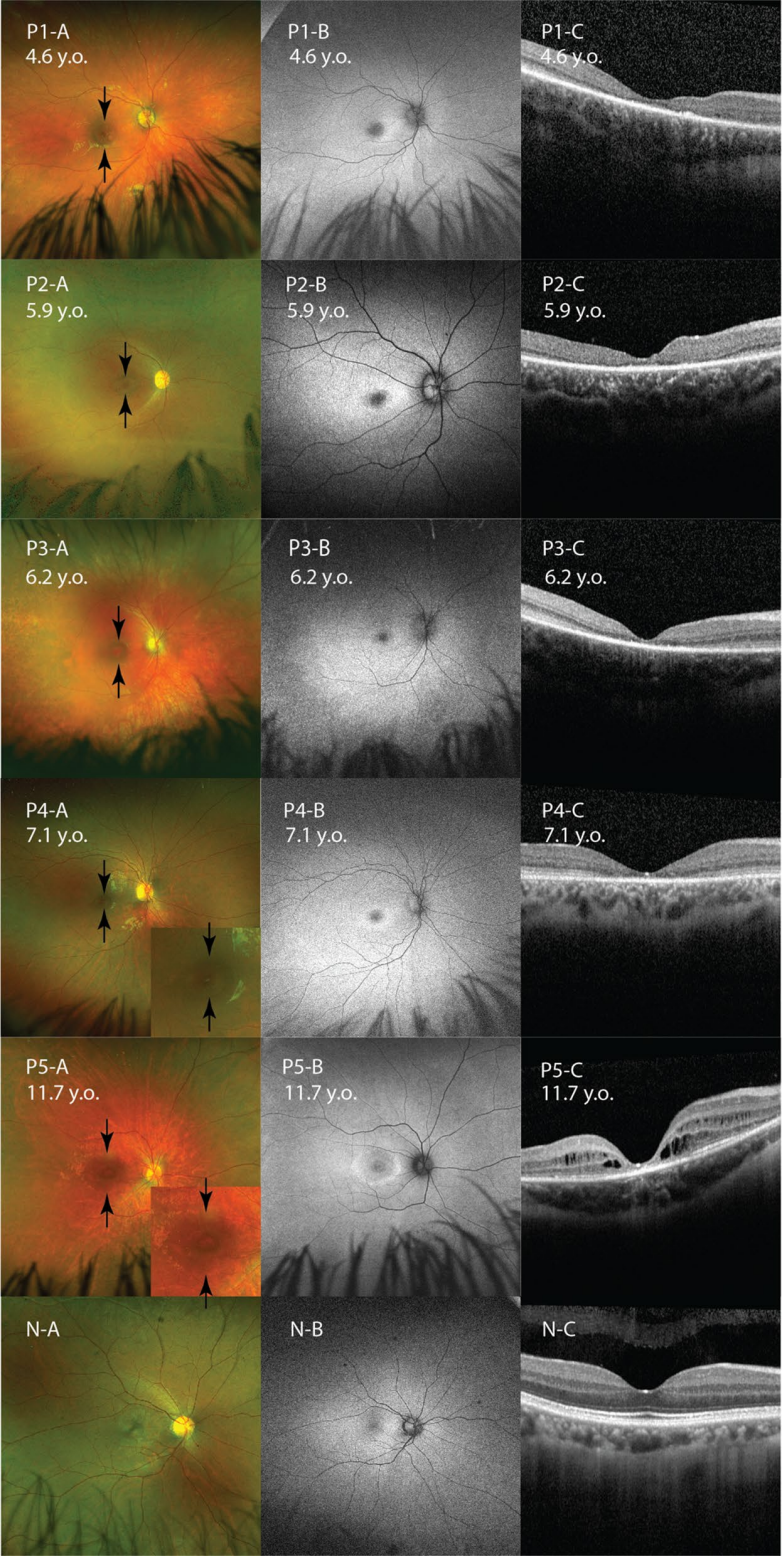
Multimodal retinal imaging for all patients. Right eye UWF-fundus pseudocolour photograph, UWF-FAF, and macular SD-OCT for P1-P5. Double black arrows indicate the margin of bull’s eye maculopathy (BEM). **(P1-A)** BEM and macular yellow-orange appearance found in P1. **(P1-B)** HyperAF ring surrounding hypoAF fovea. **(P1-C)** EZ loss on fovea. **(P2-A)** BEM was found in P2. **(P2-B)** HyperAF ring surrounding hypoAF fovea. **(P2-C)** EZ loss on fovea. **(P3-A)** BEM, macular yellow-orange appearance, vessel rarefication, retinal atrophy outside vascular arcade found in P3. **(P3-B)** HyperAF ring surrounding hypoAF fovea, hypoAF corresponding to retinal atrophy. **(P3-C)** EZ loss on fovea. **(P4-A)** BEM and vessel rarefication found in P4. Insert image of enlarged macula shows BEM. **(P4-B)** HyperAF ring surrounding hypoAF fovea. **(P4-C)** EZ loss on fovea. **(P5-A)** BEM, macular striae, macular yellow-orange appearance, vessel rarefication found in P5. Insert image shows clearer macular striae. **(P5-B)** HyperAF ring surrounding hypoAF fovea. **(P5-C)** Schitic changes on macula and EZ loss on fovea. **(N-A,B,C)** Normal control showed normal fundus pseudocolour photograph, normal UWF-FAF with foveal reduction of AF, and normal SD-OCT with normal thickness and distinct lamination. *AF* Autofluorescence, *BEM* Bull’s eye maculopathy, *EZ* ellipsoid zone, *FAF* fundus autofluorescence, *SD-OCT* spectral domain-optical coherence tomography, *UWF* ultra-wide field

**Fig. 3:**
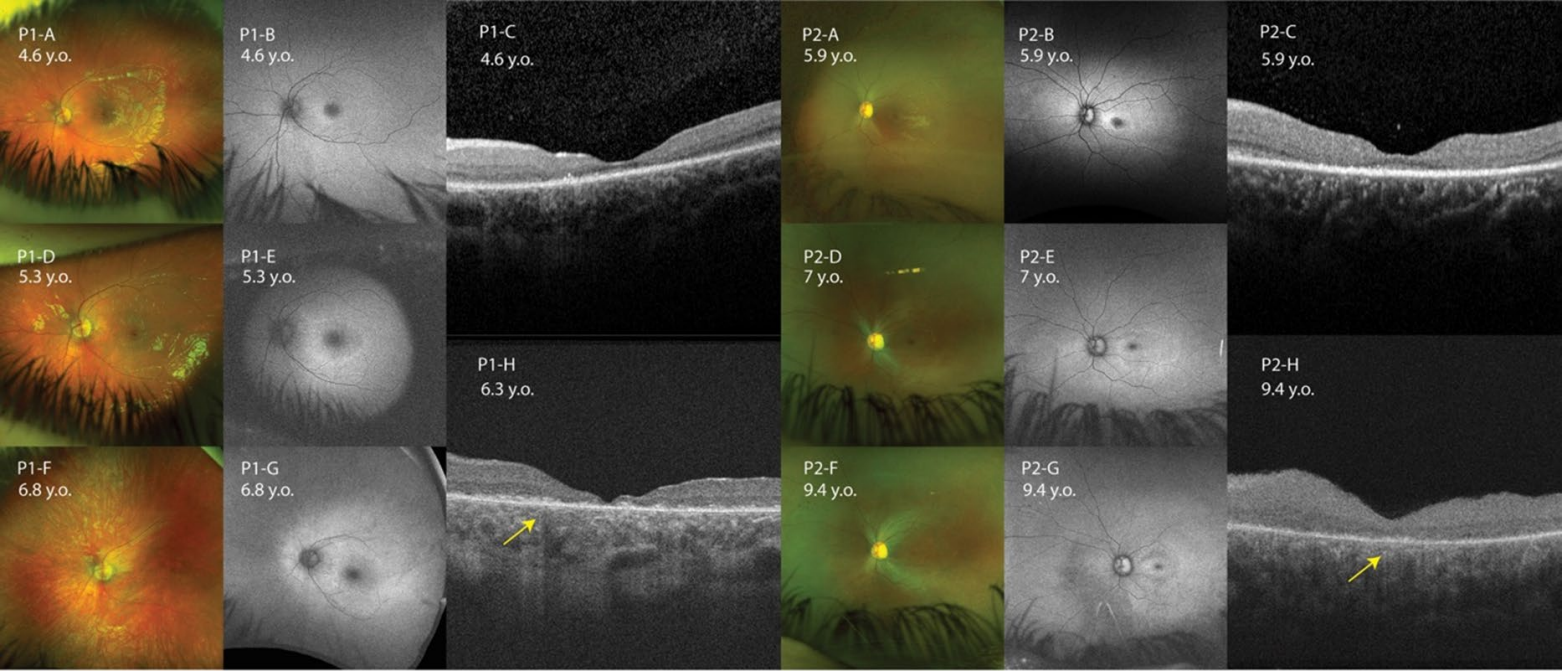
Multimodal retinal imaging follow-up for selected patients. The left eye multimodal retinal imaging was selected to illustrate change over time. UWF-fundus pseudocolour photography, UWF-FAF, and SD-OCT follow-up of P1 and P2 are shown. **(P1-A)** Bull’s eye maculopathy (BEM) and macular yellow-orange appearance were found in P1 at 4.6 yrs. **(P1-D)** Macular yellow-orange appearance became more apparent at 5.3yrs. **(P1-F)** Macular yellow-orange appearance covering macula and retinal atrophy outside vascular arcade at 6.8 yrs. **(P1-B)** HyperAF ring surrounding hypoAF fovea at 4.6 yrs. **(P1-E)** More apparent perifoveal hyperAF ring at 5.3 yrs. **(P1-G)** Perifoveal hyperAF ring disappearance, hypoAF outside vascular arcade corresponding to retinal atrophy at 6.8 yrs. **(P2-A)** BEM was found in P2 at 5.9 yrs. **(P2-D)** Macular yellow-orange appearance started to bed found at 7 yrs. **(P2-F)** BEM, macular yellow-orange appearance, vessel rarefication, retinal atrophy on inferonasal area at 9.4 yrs. **(P2-B)** HyperAF ring surrounding hypoAF fovea at 5.9 yrs. **(P2-E)** More apparent perifoveal hyperAF ring at 7 yrs. **(P2-G)** Perifoveal hyperAF ring disappearance, replaced by hypoAF ring. HypoAF on inferonasal area corresponding to retinal atrophy at 9.4 yrs BL SD-OCT was taken using Heidelberg, while FU SD-OCT was taken using Cirrus device. **(P1-C)(P2-C)** BL SD-OCT of P1 and P2 showed disruption of foveal EZ. **(P1-H)(P2-H)** FU SD-OCT of P1 and P2 showed progressed disappearance of EZ. Signal hypertransmission into choroid **(yellow arrow)** was present in both FU SD-OCT. *AF* Autofluorescence, *BEM* Bull’s eye maculopathy, *BL* baseline, *EZ* ellipsoid zone, *FAF* fundus autofluorescence, *FU* follow-up, *SD-OCT* spectral domain-optical coherence tomography, *UWF* ultra-wide field

Assessment of UWF-fundus pseudocolour appearance and UWF-FAF showed a consistent bull’s eye macular appearance in all patients (Fig. 2). Additionally, P5 showed macular striae. Progress of yellow-orange macular appearance, retinal atrophy, and vessel rarefication can be observed in P1 and P2 (Fig. 3). The FAF pattern consisted of hyper-autofluorescence (hyperAF) rings surrounding a hypo-autofluorescence (hypoAF) fovea (Fig. 2). This perifoveal hyperAF ring became more apparent at first FAF FU in P1 and P2. On the second FU, the ring of hyperAF had disappeared and hypoAF had developed outside the vascular arcade corresponding to the retinal atrophy seen on fundus image (Fig. 3). Right and left eyes of the patients showed similar phenotype.

### Spectral domain-optical coherence tomography (SD-OCT)

Foveal ellipsoid zone (EZ) disruption was found in each patient. Those with the largest central EZ disruption had the poorest eventual BCVA (P1&2) (Table 1). FU OCT was available in P1 and P2 using the Cirrus device and showed progression of EZ loss and signal hypertransmission into the choroid (Fig. 3). Macular IR appearances again showed a bull’s eye maculopathy for P1-P5 and also macular striae for P5. CSTs were ranging from 99 to 145 μm (Table 1). In the compound heterozygous *CLN3* patient (P5), we identified macular inner and outer retinal microcystic changes in addition to the macular atrophy (Fig. 2). There is a concordance in SD-OCT result of each patient.

### Electrophysiology

**Fig. 4:**
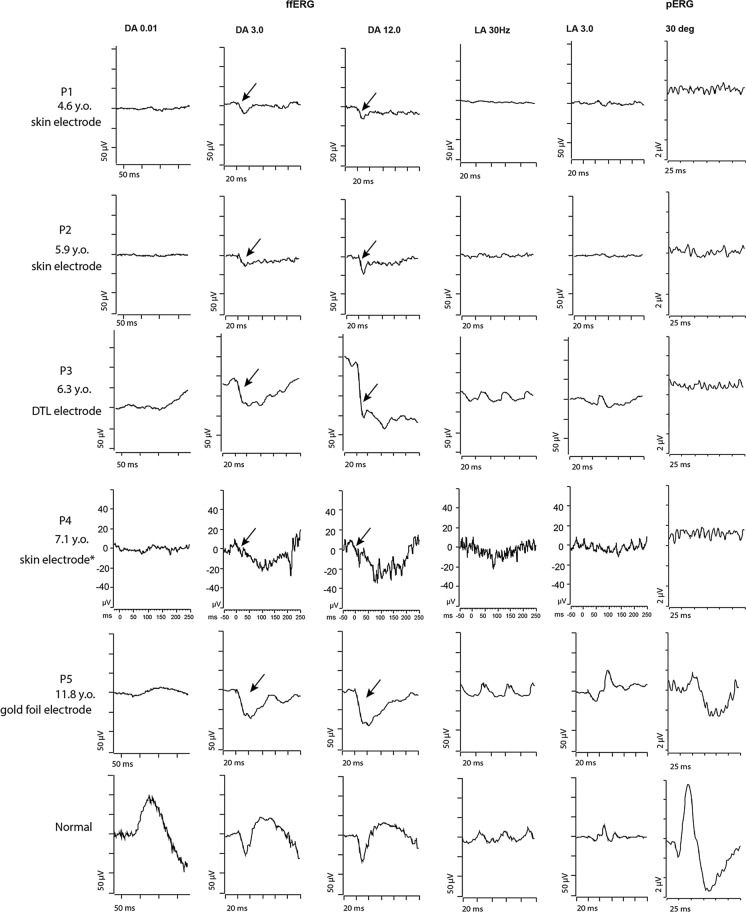
Full-field ERGs and pattern ERG recordings. The full-field ERGs were recorded according to ISCEV protocols for paediatric ERG and in one case the non-standard abbreviated ERG protocol was used. P1 and P2 were examined using skin electrodes, P3 used DTL electrodes, P4 underwent a paediatric non-standard abbreviated ERG protocol using skin electrode, and P5 used gold foil electrodes. P4 ERG was performed using a modified Great Ormond Street Hospital (GOSH) protocol as described in the methods. All patients showed severely reduced or undetectable DA 0.01 response. All patients excluding P4 showed a reduced b:a wave ratio (electronegative) for DA 3.0 and DA 12.0. P4 had a very noisy recordings, and there is a suggestion of a reduced b wave. The LA 30 Hz and LA 3.0 were significantly reduced for P1-P2. P4’s responses were noisy but also appeared reduced. Patients P3 and P5 were at the lower of the normal range. In patients P1 to P4, the pERG 30 deg p50 amplitude was almost undetectable. Patient P5 showed an identifiable waveform, but the p50 amplitude was reduced. Despite P4 having poor compliance and cooperation during the testing which resulted in noisy recordings, the combination of the potential electronegative scotopic ERG and a significantly reduced LA 30 Hz and LA 3.0 raised the possibility of Batten disease as a potential diagnosis. *DA* Dark adapted, *DTL* Dawson, Trick, and Litzkow electrodes, *ERG* electroretinogram, *ffERG* full-field ERG, *LA* light adapted, *pERG* pattern ERG

P1, P2, and P4 used skin electrode, while P3 and P5 used DTL and gold foil electrode, respectively. P4 underwent paediatric non-standard abbreviated ERG protocol with skin electrode. The electrophysiology results were as follows. The pERG recordings were noisy and almost undetectable for the 15-degree stimulus field. The 30-degree field had identifiable traces but greatly reduced. The ffERG revealed an overall electronegative ERG waveform in addition to the reduced dark adapted (DA) and light adapted (LA) responses. P2 had ERG twice with 1.1-year interval. The first one showed presence of rod function and undetectable cone response, while the second one showed worsening of rod function (Table 1) (Fig. 4).

### Associated neurobehavioural issues

Neurobehavioural issues were documented in 4 patients (P1-4) at the time of presentation. The oldest patient (P5) did not have any systemic symptoms at presentation or the last ocular follow-up. Associated symptoms included autism spectrum features in P1, significant anxiety and speech delay in P2, behavioural issues in P3, and motor dyspraxia and behavioural issues (biting friends) in P4. In two patients (P2 and P4) the neurological abnormalities appeared after the visual symptoms, while P3 had onset before any eye complaint.

## Discussion

*CLN3-*related disease commonly presents with early onset visual decline and variable neurodegeneration in childhood [12]. The visual decline in children with *CLN3* disease is frequently more rapid than other early onset maculopathies such as Stargardt disease [32].

The CLN3 protein has a crucial role within neurons specifically in the synaptic space, with animal models of *CLN3* disease showing this condition is primarily a disease of the inner retina, with secondary changes in the outer retina [17, 20]. CLN3 has a role in the transfer of the palmitoyl-protein thioesterases-1 (Ppt1) protein, and deficiencies in this protein have been associated with inner nuclear layer damage, particularly cone bipolar cells, and further damaging the cone photoreceptor cells over the rod [33, 34]. This pathophysiology assists in the understanding of the generation of the electronegative ERG, the one feature that was consistent across our cohort and similar to previous studies [18, 21, 22, 32], reflecting the inner retinal defects. There was significant but variable reduction in both rod and cone responses as found in other studies [9, 18, 21, 35]. The ffERG of P2 in 2 different time points showed early DA ERG preservation associated with an undetectable LA ERG, further reflecting initial cone involvement of this disease and thus resembling CRD [33, 34]. In contrast, other studies in *CLN3* studies in cases without neurological phenotype showed that DA ERG is more affected that LA ERG resembling RCD [8, 9, 32, 35]. These contrasting phenotypes have electronegative ERG or at least reduced b:a wave ratio as the consistent common finding reflecting inner retina disturbance.

We found the most common pathogenic *CLN3* variant of c.461-280_677 + 382del in all 5 patients [36–38]. In 4 patients (P1-P4), this variant was homozygous. In P5 we identified this common pathogenic variant in compound with a novel missense variant, c.680A > G p.(Tyr227Cys). This variant is likely pathogenic according to ACMG classification [39].

Batten disease is a rare paediatric degenerative disorder, and diagnosis may be delayed due to variable presenting features [18, 21, 40]. The application of electrophysiology combined with multimodal imaging in patients with reduced vision provides an opportunity of early recognition of this disease. The findings of an electronegative ERG and biomarkers of a bull’s eye maculopathy facilitate directed genetic testing. The increasing availability of genetic testing will supplant the use of peripheral blood film microscopy (vacuolated lymphocytes) and electron microscopy (storage lysosomal inclusions) as previously proposed by other authors [18].

Ophthalmic follow-up is challenging for these patients due to poor cooperation as the degenerative disorder progresses. In our study two patients had reliable measurements to enable comparison with BL. In these two patients (4 eyes) the rate of change was a loss of 0.75 (0.41) logMAR letters/year during 3.9 (2) years of FU. It is a slower rate of deterioration with longer FU compared to Wright et al*.* study with 2.02 (3.78) logMAR letters/year during 0.9 (0.5) years FU [18]. These results provide further evidence to the variability in disease progression in this disorder. The latest-onset patient (P5) with no documented neurological findings had the best BCVA, while the early onset patients (P1&2) had the worst BCVA at FU. P5 was the only patient with a compound heterozygous mutation. These findings were in concordance to a previous non-syndromic *CLN3* study that found absence of visual loss in the late onset patients and mild visual loss in their early onset patients [9]. Later onset of the disease appears to be correlated with better BCVA. A vast majority of *CLN3* disease patients (± 80%) present with vision impairment [41, 42]. A contribution to the visual decline has been postulated to arise from additional damage to the lateral geniculate nucleus and/or primary visual cortex [43].

Bull’s eye maculopathy is the most consistent and prominent macular finding in this patient cohort as also found in previous studies [18, 21, 44]. Other fundus findings reported in *CLN3* disease include optic disc pallor, macular atrophy, macular striae, macular oedema, retinal pigment epithelium (RPE) atrophy, RPE granularity, bone spicule formation, epiretinal membrane, arteriolar attenuation, and even a Coats-like reaction [9, 18, 40, 45]. The fundus variability may lead to misdiagnosis of Stargardt disease or retinitis pigmentosa, demonstrating the importance of electrophysiology investigations.

UWF-FAF findings highlighted the central hypoautofluorescence (hypoAF) surrounded by a ring of hyperAF found in our patients. Through 2.2–3.5 years of UWF-FAF follow-up in P1&P2, we found that the perifoveal hyperAF ring as found in previous *CLN3* study [18] became more apparent and eventually disappeared. Then hypoAF starts to emerge in the periphery corresponding to retinal atrophy [40]. As disease advances, the whole macular region shows generalised hypoAF [9, 18, 46]. Therefore, we suggest that this specific change in UWF-FAF can be used as biomarker to monitor natural disease progression. A ring of hyperAF is a common finding in rod-cone dystrophies where the ring divides healthy central retina and disturbed peripheral retina [47, 48]. Our *CLN3* cases initially show the reverse pattern with an abnormal central fovea region and preserved peripheral retina.

The disrupted foveal EZ on SD-OCTs (P1-P5) is consistent with a previous review [18] and supports the CRD phenotype reflected from ERG and UWF-FAF findings in our cohort. In contrast, *CLN3* cases with RCD phenotype had the predictably preserved foveal EZ while disrupted in the parafovea [8, 9]. In later stage, there is marked macular EZ disruption with difficulty identifying any remaining outer retinal structures and choroidal signal hypertransmission reflecting RPE disturbance [18, 46, 49, 50]. Inner and outer retinal microcystic changes found in P5 were also found in previous reports of *CLN1* and *CLN3* patients, indicating the involvement of both retinal layers [8, 9, 51]

The mechanism for retinal degeneration in *CLN3* disease is yet to be understood [43]. The bull’s eye maculopathy, early DA ERG preservation, pERG disturbance, and foveal EZ disruption in our study support the notion that this disease has centrifugal (central to peripheral) progression as also found by Preising et al*.* in their study [52]. This condition primarily affects the inner retina with secondary defects in outer retina, as suggested in a mouse model where there were significant bipolar cell survival and preserved retinal function after gene therapy [20].

Four of our patients (P1-4) had neurological problems co-existing with their ocular symptoms, while the oldest patient (P5) did not have any systemic symptoms at presentation or the last ocular follow-up. Reflected by our P5 case, electrophysiology is the primary investigations in the event of a bull’s eye maculopathy in a child of this age. An electronegative ERG with bull’s eye maculopathy should directly lead to investigation of a genetic referral even in the case without neurological symptoms. Neurological onset is variable and may occur before, after, or concurrent with visual decline. Various neurological signs and symptoms have been reported, including: dementia, seizures, speech delay, mood fluctuations, difficult behaviour, balance, or memory changes, cognitive decline, sleep disturbances, feeding difficulties, clumsiness, and poor concentration [13, 18, 41] with seizure as the most common [13]. *CLN3* has a variable phenotype as illustrated by those presenting with mild or delayed neurological defects ranging from 3- to 18-year interval between ocular and neurological onset [53–56], or no systemic features [9, 10, 15]. Ocular and neurological phenotypic variability also is frequently reported in those with the same mutations [57–59]. Ocular phenotype variability includes RCD and CRD [15]. *CLN3* literature implies that syndromic CLN3 disease (mostly homozygous variant) is characterized by CRD with childhood onset and rapid disease progression, while the isolated retinal degeneration case (mostly compound heterozygous variant) is rather a RCD with later onset and slower progression [32]. However, genotype–phenotype correlation in *CLN3* disease is not perfect and caution should be given in establishing the diagnosis [8].

Although there is no current definitive treatment for *CLN3* disease, early diagnosis is important to give appropriate family counselling and establish supportive therapies at the earliest opportunity [20, 60–76]. In Australia there is Mackenzie’s mission a study investigating preconception for autosomal recessive disorder. *CLN3* is one of the gene of 500 genes in the panel for both parents. Secondly, whole genome screening is being investigated as an expansion of the newborn screening programme to identify and enable early management of severe genetic diseases [77, 78]. There are currently 3 active *CLN3* clinical trials which have ophthalmic parameter measurement as an endpoint. These include intrathecal gene therapy AT-GTX-502 (NCT03770572), oral drug PLX-200/gemfibrozil (NCT04637282), and oral drug Miglustat 100 mg (NCT05174039) which give hope that disease-modifying therapies are emerging [79]. Those studies emphasize the importance of understanding the ocular biomarkers in *CLN3* disease natural history. Multimodal imaging results are similar between the two eyes in each patient, making it viable to use fellow eye as control in the event of intraocular therapeutical trials. Combination of therapies might be needed to treat this condition [73, 80, 81].

Given the retrospective nature of our study and the natural history of neurodegenerative decline in *CLN3* patients, there were limitations of follow-up examinations.

## Conclusions

The findings of an electronegative ERG with concurrent bull’s eye maculopathy in young age should prompt early neurological assessment for signs of neurodegeneration and referral for genomic investigation for *CLN3* gene defects. Some children also experience isolated ocular presentations without neurobehavioral features. It is important that *CLN3* disease is considered in electronegative ERG-bull’s eye maculopathy patients even without neurological defect. Recognition of these features will assist in establishing an early diagnosis enabling appropriate therapies, family planning, disease monitoring, and potential enrolment in clinical trials for novel therapies.

Monitoring visual function is challenging in this cohort as neurological deterioration progresses. Finding ocular biomarkers that can be consistently recorded in an outpatient setting is important for clinical trial outcome measures. Given their change throughout the natural history of the disease, EZ and FAF are the most promising structural parameters identified in our cohort.

## Declarations

### Statement on the welfare of animals

This research did not involve animals.
